# Management of obstructive sleep apnea in edentulous patients: an overview of the literature

**DOI:** 10.1007/s11325-015-1285-9

**Published:** 2015-11-19

**Authors:** David S. P. Heidsieck, Maurits H. T. de Ruiter, Jan de Lange

**Affiliations:** Department of Oral and Maxillofacial Surgery, Suite A1-121, Academic Medical Center, University of Amsterdam, Meibergdreef 9, 1105 AZ Amsterdam, The Netherlands

**Keywords:** Obstructive sleep apnea, Edentulism, Oral appliance therapy, Dentures

## Abstract

**Purpose:**

A high prevalence of obstructive sleep apnea (OSA) is seen in edentulous individuals. Treatment options for edentulous OSA patients however are limited with continuous positive airway pressure therapy (CPAP) remaining the current therapy of choice. As CPAP is associated with high non-adherence rates and oral appliance therapy requiring sufficient dentition, there is a clinical need for effective treatment strategies aimed at edentulous OSA patients. The purpose of this study was to present a thorough overview of the literature regarding (1) the effects of nocturnal denture wearing on OSA, (2) the outcomes of oral appliance therapy, and (3) surgical treatment in edentulous OSA patients.

**Methods:**

A computer-assisted literature search was performed in the MEDLINE database on “edentulism” and “obstructive sleep apnea.” The search yielded a total of 34 original articles.

**Results:**

A total of 20 studies were included after exclusion of non-relevant, duplicate, and non-English publications, comprising 4 randomized clinical trials, 12 case reports, and 4 cohort or cross-sectional studies. The outcomes of these studies were addressed in detail concerning nocturnal wearing of dentures, oral appliance therapy, and surgical treatment.

**Conclusion:**

Currently, there is no consensus in the literature on the effects of nocturnal wearing of dentures on OSA. Several studies report the successful use of oral appliance therapy, including implant-retained mandibular advancement devices (MADs), in selected cases of edentulous patients with varying stages of OSA. Little evidence is available regarding outcomes of surgical procedures in edentulous patients. Based on the results of this literature overview, the paucity of effective evidence-based treatment strategies for edentulous OSA patients indicates the further need of clinical studies to improve clinical management.

## Background

Obstructive sleep apnea (OSA) is a major medical problem, estimated to affect up to 15–30 % of male adults and up to 5–15 % of female adults [1, 2]. It is characterized by repetitive obstructions of the upper airway during sleep, frequently causing oxygen desaturation. This induces repeating arousals resulting in fragmented sleep and excessive daytime sleepiness. Untreated OSA can lead to a number of complications including cardio- and cerebrovascular disease [3, 4], diabetes mellitus [5], impaired cognitive functions [6], and depression [7]. Additionally, OSA patients are more likely to be involved in motor vehicle crashes [8] and have an increased risk of all-cause mortality compared to individuals without OSA [9].

The underlying pathophysiology of OSA may vary by age, with increased upper airway collapsibility being a common cause in older patients [10]. Previous studies revealed that patients suffering from edentulism are more likely to develop OSA [11, 12]. The absence of dentition causes a loss of vertical dimension and is associated with morphological changes in the upper airway, decrease of the retropharyngeal space, and decrease in size and tone of the pharyngeal musculature [13, 14]. Despite advances in primary dental care, edentulism remains a common condition in senior adults, with incidence rates between 3 and 80 % depending on the country of residence [15, 16].

Current guidelines propose non-surgical treatment options including lifestyle adjustments, position therapy, and (nasally applied) continuous positive airway pressure (CPAP) as first-line therapy. Second in line are treatments with oral appliances or surgical approaches based on the degree of severity and etiology [17]. Edentulism considerably reduces the number of available treatment options, with CPAP generally being the treatment of choice. While the efficacy of CPAP is reliant on patient adherence, non-adherence rates ranging from 29 to 83 % are reported, with many patients rejecting therapy within the first months after initiation. Issues with mask discomfort, nasal dryness or congestion, and difficulty adapting to the pressure have been identified as barriers to patient compliance in CPAP therapy [18, 19]. Although oral appliance therapy has proven to be an effective non-invasive therapy suitable for patients with mild to moderate OSA, they generally are not indicated for edentate patients as sufficient dentition is required to support and retain the appliance [20]. Petit et al. reported that in up to 34 % of all OSA cases, oral appliance therapy is contraindicated due to dental limitations [21]. Although other therapies including soft tissue surgery or maxillomandibular advancement (MMA) surgery can be considered by clinicians to treat more severe cases of OSA, few evidence is reported in the literature regarding outcomes of such procedures in edentulous OSA patients.

As treatment of edentulous OSA patients can be challenging, all available treatment options should be considered to ensure optimal management and prevent possible life-threatening complications. The present study aims to provide a thorough overview of the literature regarding (1) the effects of nocturnal denture wearing on OSA, (2) the outcomes of oral appliance therapy, and (3) surgical treatment in edentulous OSA patients.

## Methods

A computer-assisted search was performed in the medical database MEDLINE (from January 1966 to March 2015). A combination of the following medical subject headings (MESH) were used in the search: “Sleep Apnea, Obstructive,” “Jaw, Edentulous,” “Jaw, Edentulous Partially,” and “Mouth, Edentulous.” Inclusion criteria consisted of studies reporting clinical outcomes on treatment of OSA in either partially or fully edentulous patients. Clinical outcome parameters were considered relevant when comprising results from polysomnography studies (PSG) or standardized sleep-related questionnaires. Excluded were studies not reporting on partially or fully edentulous patients, studies lacking relevant clinical outcomes, review articles, non-original studies, and articles written in other languages than English. Potential relevance of the articles was determined by studying their titles and abstracts. When considered relevant, full texts were obtained and read; other studies were included by screening the reference lists of articles that were found during our original search. Relevant data regarding patient characteristics, treatment, and clinical outcome including age, gender, body mass index (BMI), polysomnography results, and questionnaire results were extracted. Included studies were appraised according to the Oxford Centre for Evidence-Based Medicine levels of evidence (Table 1) [22].

**Table 1:** Oxford Centre for Evidence-Based Medicine levels of evidence

Level	Therapy
1a	Systematic review of randomized controlled trials
1b	Individual randomized controlled trial
2a	Systematic review of cohort studies
2b	Individual cohort study
2c	“Outcomes research”
3a	Systematic review of case–control studies
3b	Individual case–control study
4	Case series (with or without comparison)
5	Expert opinion

## Results

Our search yielded a total of 34 articles. One study was excluded for being retracted from the respective journal, 1 article reported duplicate findings, and 11 articles did not report clinical outcomes or were non-relevant to the topic. Another study published in a language other than English was excluded. No review articles were identified. The remaining 20 studies, comprising 4 clinical trials, 12 case reports or case series, 3 cross-sectional studies, and 1 cohort study, were included for further examination.

The results and outcomes of all included studies are summarized in Tables 2, 3, and 4 and will be discussed from here on. Table 2 provides an overview of clinical studies investigating the effects of nocturnal wear of dentures on OSA. Studies reporting treatment of edentulous OSA patients with oral appliances are summarized in Table 3. Table 4 summarizes studies reporting surgical treatment of edentulous OSA patients.

**Table 2:** Clinical studies reporting nocturnal wearing of dentures in edentulous patients

Author	Year	Study type	Level of evidence	Subjects	No.	Patient characteristics			Duration/follow-up	Sleep study	Results (with dentures vs. without dentures)			Conclusion (effects of nocturnal denture wearing)
						Age (years)	Gender (M/F)	BMI (kg/m^2^)			AHI (/h)	Mean SaO_2_ (%)	Lowest SaO_2_ (%)	
Bucca et al. [11]	1999	Case series	4	Edentulous OSA patients	*N* = 6	63 ± 4	M: *n* = 6	31 ± 2	2 × 1 night (consecutive)	In-laboratory PSG	13 ± 4 vs. 20 ± 5	92 ± 2 vs. 90 ± 2	85 ± 2 vs. 82 ± 2	(1) Decrease of AHI (2) Increased mean/lowest SaO_2_ (3) Increase of retropharyngeal space
Bucca et al. [12]	2006	Clinical trial	2b	Edentulous OSA and non-OSA patients	*N* = 48	69 ± 9	M: *n* = 29 F: *n* = 19	28 ± 1	2 × 1 night (consecutive)	In-laboratory PSG or ambulatory PSG	11 ± 2 vs. 17 ± 4	93 ± 0 vs. 93 ± 1	84 ± 1 vs. 82 ± 1	(1) Decrease of AHI (2) Increased mean/lowest SaO_2_ (3) Decreased exhaled eNO (4) Increase of retropharyngeal space
Almeida et al. [26]	2012	Randomized clinical trial	2b	Edentulous OSA patients, age ≥ 60 years	*N* = 23	70 ± 5	M: *n* = 6 F: *n* = 17	27 ± 3	2 × 15+ days	In-laboratory PSG	26 ± 15 vs. 20 ± 10	NS	NS	(1) Increase of AHI (2) No significant difference in mean/lowest SaO_2_
Arisaka et al. [27]	2009	Clinical trial	2b	Edentulous OSA and non-OSA patients	\*N* = 34	73 ± 9	M: *n* = 16 F: *n* = 18	23 ± 4	2 × 1 night (consecutive)	Ambulatory PSG	13 ± 10 vs. 18 ± 15	NS	NS	(1) Decrease of AHI in 19/27 patients (2) Increase of AHI in 8/27 patients (3) No significant difference in lowest/mean SaO_2_

Data regarding age, BMI, AHI, and mean and lowest hemoglobin oxygen saturation (SaO_2_) are presented as mean ± standard deviation

*AHI* apnea–hypopnea index, *eNO* exhaled nitric oxide (marker for inflammation), *NR* not reported, *NS* not significant, *OSA* obstructive sleep apnea, *PSG* polysomnography, *SaO*_*2*_ hemoglobin oxygen saturation

**Table 3:** Studies reporting oral appliance treatment in edentulous OSA patients

Author	Year	Study type	Level of evidence	Study group	No.	Patient characteristics				Type of appliance	Fixation	Follow-up	Results (baseline vs. OA therapy)			Conclusion (effects of OA therapy)
						Age (years)	Gender (M/F)	BMI (kg/m^2^)	OSA type				AHI (/h)	Mean SaO_2_ (%)	Lowest SaO_2_ (%)	
Eskafi et al. [41]	2004	Clinical trial	4	OSA patients with CHF—6/25 with (partial) dentures	*N* = 25	66 ± 8^a^	M: *n* = 24F: *n* = 1^a^	26 ± 4^a^	Mild to moderate^a^	MAD (Orthocryl)	Existing dentition or upper and/or lower dentures	4–5 weeks	19 ± 12 vs. 12 ± 10^a^	NR	NR	(1) Decrease of AHI (pooled results from entire group)
Giannasi et al. [42]	2008	Case report	5	Maxillary edentulous OSA patient	*N* = 1	46	M	29	Moderate	MAD (PM Positioner)	Maxillary denture/mandibular dentition	6 months	18 vs. 2	NR	83 vs. 92	(1) Decrease of AHI (2) Increased SaO_2_
Giannasi et al. [43]	2010	Case report	5	Partial maxillary and mandibular edentulous OSA patient	*N* = 1	74	M	28	Moderate	MAD (PM Positioner)	Remaining dentition	6 months and 2 years	19 vs. 8 vs. 8	NR	80 vs. 86 vs. 86	(1) Decrease of AHI (2) Increased SaO_2_
Ogawa et al. [44]	2011	Case report	5	Partial maxillary and mandibular edentulous OSA patient	*N* = 1	58	M	23	Moderate	MAD (self-fabricated)	Remaining dentition	2 weeks	22 vs. 8	93 vs. 99	83 vs. 88	(1) Decrease of AHI (2) Increased SaO_2_
Keyf et al. [45]	2014	Case report	5	Mandibular edentulous OSA patient	*N* = 1	63	F	25	Mild	MAD (Monoblock)	Mandibular ridge/maxillary dentition	5 months	13 vs. 3	95 vs. 93	75 vs. 88	(1) Decrease of AHI (2) Increased SaO_2_
Piskin et al. [46]	2010	Case report	5	Completely edentulous OSA patient with bulky masseter muscles	*N* = 1	61	F	NR	Severe	MAD (modified for masseter muscle lateralization, Orthocryl)	Mandibular and maxillary ridge	1 month	98 vs. 15	81 vs. 94	69 vs. 82	(1) Decrease of AHI (2) Increased SaO_2_
Nelogi et al. [47]	2011	Case report	5	Completely edentulous OSA patient	*N* = 1	61	M	NR	Mild	MAD (self-fabricated)	Upper and lower dentures	NR	12 vs. 4	NR	NR	(1) Decrease of AHI
Kurtulmus et al. [48]	2009	Case report	5	Completely edentulous OSA patient, retrognatic mandible, tongue base collapse at rest	*N* = 1	56	F	NR	Mild	Mandibular and tongue advancement splint (self-fabricated)	Tongue and mandibular/maxillary ridge	NR	8 vs. 4	NR	83 vs. 96	(1) Decrease of AHI (2) Increased SaO_2_
Hoekema et al. [49]	2007	Case series	4	Edentulous OSA patients	*N* = 6	56 ± 5	M (*n* = 6)	27 ± 3	Mild to moderate	MAD (modified Thornton adjustable positioner)	Endosseous implants	6–24 months	–^b^	NR	–^b^	AHI < 5 reached in 4/6 patients with treatment
de Carlos et al. [50]	2010	Case report	5	Completely edentulous OSA patient	*N* = 1	49	M	29	Moderate	Inter maxillary elastic bands	Ortho-implants	2 months	25 vs. 2	NR	87 vs. 92	(1) Decreased AHI (2) Increased SaO_2_

Data regarding age, BMI, AHI, and mean and lowest hemoglobin oxygen saturation (SaO_2_) are presented as mean ± standard deviation

*AHI* apnea–hypopnea index, *CHF* congestive heart failure, *F* female, *M* male, *MAD* mandibular advancement device, *NR* not reported, *NS* not significant, *OA* oral appliance, *OSA* obstructive sleep apnea, *SaO*_*2*_ hemoglobin oxygen saturation

^a^Pooled data from entire cohort (study did not report separate data of the edentulous subgroup)

^b^This study reported individual patient results only

**Table 4:** Studies reporting surgical treatment in edentulous OSA patients

Author	Year	Study type	Level of evidence	Study group	No.	Patient characteristics^a^				Surgical procedure	Follow-up	Outcomes (pre- vs. posttreatment)^a^			Conclusion (effects of surgical treatment)
						Age (years)	Gender (M/F)	BMI (kg/m^2^)	OSA type			AHI (/h)	Mean SaO_2_ (%)	Lowest SaO_2_ (%)	
Alvarez et al. [54]	1987	Case report	5	Completely edentulous OSA patient. Medical history: UPPP,tracheostomy, and rhinoplasty	*N* = 1	59	M	NR	Severe	Mandibular advancement and genioplasty	3 months	NR	NR	NR	(1) Free of apneic events posttreatment (117 vs. 0)
Sencimen et al. [55]	2012	Case report	5	Maxillary and partial mandibular edentulous OSA patient	*N* = 1	49	M	32	Severe	Maxillomandibular advancement surgery	9 months	82 vs. 1	90 vs. 92	74 vs. 80	(1) Decreased AHI (2) Increased SaO_2_

Data regarding age, BMI, AHI, and mean and lowest hemoglobin oxygen saturation (SaO_2_) are presented as mean ± standard deviation

*AHI* apnea–hypopnea index, *BMI* body mass index, *OSA* obstructive sleep apnea, *SaO*_*2*_ hemoglobin oxygen saturation, *UPPP* uvulopalatopharyngoplasty

### Nocturnal use of dentures

Edentulism is associated with anatomical changes and it is associated with an increased prevalence of OSA [12]. Dentures are intended to restore this natural anatomy and are being recognized to cause changes in the mandible, tongue, soft tissue, and the pharyngeal airway space [23]. As a result of this, wearing dentures during sleep has been proposed to promote an increase in the retroglossal space and prevent or reduce OSA in edentulous patients [11, 12].

With a study of six edentulous OSA patients, Bucca et al. [11] were the first authors to report the relationship between edentulism, nocturnal denture wearing, and OSA. In patients not wearing their dentures overnight, the authors found a significant worsening of apnea–hypopnea index (AHI) and nocturnal oxygen saturation (SaO_2_) levels, as well as a reduction of the retropharyngeal space. Wearing complete dentures overnight showed to improve both AHI and SaO_2_ levels and resulted in an increase in the retropharyngeal space.

Similar findings were found in a larger clinical trial evaluating PSG results and cephalometric exams (CE) of 48 edentulous individuals with complete dentures [12]. In this study, participants were likewise monitored during sleep on two consecutive nights, one night with dentures and one night without. PSG examinations in this study were taken either at home or in-hospital. Almost half of the participants met the criteria for OSA, while the remaining participants did not appear to have any sleep-related breathing disorder. An improvement of AHI and mean and lowest SaO_2_ was seen in both OSA and non-OSA patients when wearing complete dentures during sleep. These positive effects appeared to be more present in non-OSA patients, suggesting the lack of dentition might play an essential causal role in the AHI increase in these individuals. CE performed in supine position displayed an increase in retropharyngeal space when patients were wearing their dentures. Also, a significant decrease in measured exhaled nitric oxygen (eNO), a marker for inflammation, was found in patients after sleeping with dentures compared to sleeping without dentures. Though the exact role of inflammation in the presence of OSA is still unclear, previous research revealed that pharyngeal inflammation was present in patients with OSA [24, 25].

The studies mentioned above performed by Bucca et al. [11, 12] indicate that nocturnal wearing of dentures could be helpful in reducing OSA in edentulous individuals. Other studies however report conflicting results or found positive effects in only the minority of patients. Almeida et al. [26], for example, reported a significant increase in apneic events in patients wearing their dentures overnight. In their clinical trial, 23 OSA patients received two in-laboratory PSG examinations: one with dentures and one night without. Overall, a worsening of AHI was reported in patients wearing their dentures during the night. In patients with mild OSA (AHI 5–15), a significant worsening of AHI was found while wearing dentures overnight, especially when sleeping in supine position. The authors note however that no significant difference was found in patients with moderate (AHI 15–30) or severe (AHI > 30) OSA, with some patients showing an improvement of AHI when wearing dentures overnight. Also, no significant differences were reported on nocturnal SaO_2_ levels in patients wearing dentures overnight compared to no denture wearing.

Arisaka et al. [27] also reported that nocturnal use of dentures does not assure an improvement of PSG parameters in all patients. In a similar trial with 27 edentulous elderly using portable sleep recording, 19 patients showed to benefit from nocturnal wearing of dentures, while in 8 patients, a worsening of AHI was observed when wearing their dentures overnight.

Several non-experimental studies have examined the relationship between nocturnal wearing of dentures and OSA as well. In a cross-sectional study of 58 elderly using questionnaires and portable sleep recordings, Endeshaw et al. [28] found a relationship between an AHI greater than 15 and denture use, with the majority of patients in their cohort not wearing their dentures overnight. In another cross-sectional study of 173 edentulous elderly, Emami et al. [29] used standardized questionnaires including the Pittsburgh Sleep Quality Index (PSQI), Epworth Sleepiness Scale (ESS), and Karolinska Sleepiness Scale (KSS) to assess the overall sleep quality and daytime sleepiness in elderly. However, no significant difference in sleep quality or daytime sleepiness was found in elderly who preferred to wear their dentures overnight compared to elderly who did not. A cohort study assessed the effects of nocturnal wear of dentures with a follow-up period of 1 year. Again, no significant differences in sleep quality or daytime sleepiness were found [30]. In another cross-sectional study using self-reported enquiries, Tsuda et al. [31] found no evidence that nocturnal denture wearing increased or reduced the risk of sleep disordered breathing.

### Oral appliance therapy

Oral appliance therapy is considered an effective treatment option for patients with mild to moderate OSA and a feasible alternative for patients intolerant to CPAP treatment [18, 32]. Among these devices, the mandibular advancement device (MAD) is the most commonly used and studied appliance [33]. MADs cover the upper and lower dentition and maintain the mandible in a protruded position during sleep. By enlarging the upper airway and reducing upper airway collapsibility (e.g., by improving upper airway muscle tone), these devices help in sustaining upper airway patency [34, 35]. In awake OSA patients, MAD insertion significantly increases electromyogram (EMG) activity of the genioglossus, geniohyoid, and masseter muscles [36].

Due to a lack of dentition to support and retain a MAD, these devices are considered unsuitable for edentulous OSA patients. If sufficient dentition is available, other contraindications to consider include temporomandibular joint (TMJ) dysfunction, severe dental mobility, and periodontal diseases [20, 21]. When MAD treatment is initiated, possible side effects that could hamper compliance include excessive salivation and poor retention on the short term and TMJ pain and myofacial discomfort on the long term [37].

Tongue retaining/reposition devices (TRDs) do not require support from dentition and can be used as an alternative in patients lacking sufficient dentition. TRDs use suction forces to hold the tongue in a forward position without mandibular repositioning and have been reported to have a similar performance to that of MADs [38]. Deane et al. investigated the efficacy of TRDs compared to MAD therapy in a randomized controlled trial and reported a similar effectiveness for both devices in terms of AHI reduction. However, side effects including soft tissue irritation, dryness of mouth, and excessive salivation were reported in the majority of patients treated with a TRD, with MAD therapy showing a substantial higher compliance and patient preference [39]. In a small study comparing three types of oral appliances, Barthlen et al. even reported no improvement of AHI in patients treated with a TRD [40].

Several case studies report the use of oral appliances in both partial and completely edentulous patients with OSA. Eskafi et al. [41] performed a clinical trial assessing the effects of MAD treatment in OSA patients with stable congestive heart failure (CHF). Six out of 25 participating patients in their study had removable dentures (4 patients with complete dentures, 2 patients with partial dentures) on which a MAD was fixed. Pooled data of all patients showed a significant improvement of AHI compared to pretreatment AHI. No specific analysis on the group of patients wearing dentures however was mentioned. Besides loosening of a fixed partial denture in the upper jaw of one patient after 6 months of treatment, no specific complications regarding the use of the devices in edentulous patients were reported. Overall, 10 patients reported complaints related to MAD use including TMJ pain, soreness of dentition, and tiredness of the jaws. These adverse effects became less intense during the course of the study.

Giannasi et al. [42] applied an adjustable MAD on a partial edentulous OSA patient with a dentate mandible. The patient’s pretreatment AHI of 18 decreased to 2/h posttreatment, and no complaints regarding wearing discomfort were mentioned. In a second case report [43], results were described regarding the use of an adjustable MAD in a partial edentate patient with moderate OSA. In their study, the MAD was fixed to remaining dentition in both jaws, resulting in a significant improvement of nocturnal AHI and SaO_2_ levels. A 2-year follow-up showed no deterioration in periodontal and gingival health. Ogawa et al. [44] also reported an improved AHI after MAD treatment of a patient with moderate OSA, missing multiple teeth and harboring severe dental problems. No side effects related to the treatment occurred during the 3-year follow-up. The authors did mention that periodical dental care was required to maintain proper oral health. Keyff et al. [45] used MAD therapy in a patient with mandibular edentulism suffering from mild OSA resulting in improvement of AHI and SaO_2_ levels. No problems were reported regarding retention or stability of the device.

The use of MAD therapy in a completely edentulous patient with severe OSA refusing CPAP treatment was reported by Piskin et al., resulting in a large improvement of AHI (98 vs. 15/h) and SaO_2_ levels [46]. A modified MAD was used to provide mandibular advancement as well as lateral displacement of the patient’s bulky masseter muscles, providing more space for the tongue. The authors mentioned a small increase in salivation and discomfort due to full-night usage of the MAD. Nelogi et al. [47] also reported MAD treatment in a completely edentate patient, in this case suffering from mild OSA. An adjusted MAD was fixed on the existing removable lower and upper dentures, resulting in improvement of AHI levels. The authors reported no complaints of discomfort during the 24-week follow-up. Kurtulumus et al. [48] used a self-fabricated mandibular and tongue advancement splint in a completely edentulous patient with mild OSA. The authors reported improvement of both AHI levels (8 vs. 4/h) and nocturnal SaO_2_. No difficulties regarding stability and usage of the device were reported.

The use of dental implants has been proposed to improve retention, as poor device retention remains a major obstacle of oral appliance treatment in edentulous patients [21, 32]. Hoekema et al. [49] reported a series of 6 edentulous patients with mild to moderate OSA who were treated with endosseous implant-retained MADs. The MADs were fixed to the mandible with a bar construction and clip attachment fixture on four dental implants that were placed in the mandible. The upper part was fixed on the maxillary ridge using suction forces. An improvement of AHI and SaO_2_ levels was notable in all 5 patients who completed follow-up, and treatment resulted in eliminating the OSA (AHI < 5) in 4 patients. Wearing discomfort was reported in only 2 patients due to excessive pressure of the MAD on the labial mucosa in the maxilla. The authors suggested to resolve such issues of discomfort with additional implant placement in the maxilla. In the single patient that underwent such a secondary treatment, complaints of wearing discomfort were resolved. This pilot study indicates that implant-retained MAD therapy could be a viable treatment option for completely edentulous OSA patients by evading poor retention and wearing discomfort.

A different intraoral approach was suggested by de Carlos et al. [50], involving the use of ortho-implants as an alternative to patients intolerant to CPAP and lacking sufficient dentition. In their case study, orthodontic elastic rubbers were anchored to two ortho-implants placed in the maxilla and two implants in the mandible of a completely edentate patient with mild OSA. Wearing of the elastics provided advancement of the mandible and resulted in an AHI drop from 25 to 2/h as well as improved nocturnal SaO_2_ levels. No problems occurred during the 8-month follow-up; however, the authors did mention the risk of loss of the ortho-implants due dynamic forces.

### Surgical treatment

Several soft tissue surgical procedures like uvulopalatopharyngoplasty (UPPP) and radiofrequency ablation (RFA) are available for OSA patients, although accompanied by varying success rates [51, 52]. In patients with severe OSA intolerant to CPAP or in whom oral devices have been found ineffective, maxillomandibular advancement (MMA) surgery has favorable efficacy regarding surgical treatment options [17, 53]. No studies could be identified reporting results of soft tissue surgical procedures in edentate patients. Limited evidence is available regarding treatment and morbidity outcomes of MMA surgery in the edentate patient.

More than two decades ago, Alvarez et al. [54] reported results of a mandibular advancement and genioplasty in an edentulous OSA patient with a medical history of UPPP, temporary tracheostomy, and rhinoplasty. Intermaxillary fixation was performed using transantral K-wire-fixed maxillary dentures and circummandibular wire-fixed mandibular dentures. No peri- or postoperative complications were reported, besides wound dehiscence, which was resolved with removal of one screw in the right ramus. Three months after the MMA procedure, the patient was free of apneic events. The authors however did not report AHI or nocturnal SaO_2_ values. A more recent case study of Sencimen et al. [55] also reported MMA surgery in a patient with complete maxillary and partial mandibular edentulism suffering from severe OSA. Temporary intermaxillary fixation was performed with wire-secured maxillary and mandibular dentures anchored to the lower residual ridge and palatinal bone with titanium mini screws. Although this patient did not have an atrophic maxilla, no intraoperative complications were reported, nor were there any other complications mentioned. Treatment resulted in an AHI drop from 82 preoperatively to 1/h postoperatively as well as improved nocturnal SaO_2_ levels.

## Discussion

Previous studies indicate that edentulous individuals are at high risk of developing OSA [11, 14]. While denture wearing may help restore the natural anatomy in edentulous OSA patients, the effect of overnight denture wearing on OSA remains controversial. Bucca et al. [11, 12] reported a significant improvement of PSG parameters in individuals wearing dentures overnight, while Almeida et al. [26] reported a worsening of these parameters after conducting a similar study. Results from Almeida et al. however showed no significant effect in patients having moderate or severe OSA, with some patients even showing an improvement of PSG parameters. To explain these findings, the authors suggested that overnight denture wearing might interrupt activation of the muscles responsible for pulling the mandible up and forward, thereby decreasing the pharyngeal patency. In patients with severe OSA, the ability to maintain appropriate muscle tone may already have been lost. These patients therefore may not experience interruption of muscle activation but receive muscle stimulation from wearing dentures, thus increasing pharyngeal patency. The authors however noted that their results may be limited since a higher average BMI was present in the mild OSA group compared to the moderate to severe OSA group.

Although the variance in the use of portable sleep recordings instead of in-laboratory PSGs among studies might have played a role in the conflicting study results, several studies confirm that ambulant PSGs are a reliable alternative for in-laboratory PSGs [56, 57].

Despite the fact that the majority of edentate patients wear their dentures overnight [58], it is generally not recommended by prosthodontists as it can result in denture stomatitis and traumatic ulcers due to pressure on the soft tissues [59]. Taking into account the potential oral health risks and current absence of conclusive evidence, clinicians treating edentate patients with OSA should be reserved in endorsing patients to wear their dentures at night.

Various treatment options have been proposed to treat OSA, ranging from oral appliance therapy to surgical interventions [17]. Morphological alterations in the edentate patient however may hamper the outcome of or compliance to these interventions when applied in edentulous patients. By addressing the major concern of poor retention due to insufficient dentition, implant-retained MADs appear to be a promising treatment option in patients with mild to moderate OSA. Excessive pressure of the MAD on the maxillary alveolar ridge however was related to wearing discomfort and reduced maximal mandibular advancement [49]. In the pilot study of Hoekema et al., these complaints were resolved by the placement of maxillary dental implants. The increased rate of alveolar bone resorption in edentate patients however should be taken into consideration, as this limits the ability of maxillary implant placement and retention [60]. Similar concerns are present in surgical treatment with ortho-implants [50] as increased bone resorption could compromise treatment success due to implant loss. These risks are also present in other procedures. Although considered a rare complication during MMA surgery in dentate patients, a high incidence of fractures have been described in the atrophic edentulous maxilla following LeFort 1 osteotomies. These fractures mostly involved the junction between the horizontal plate of the palatine bone and posterior part of the maxilla [61]. Such complications can eventually lead to aseptic necrosis and surgeons should bare that in mind when considering this intervention [62].

It should be noted that OSA patients in general have an increased risk of postoperative complications including postoperative desaturation, respiratory failure, postoperative cardiac events, and ICU transfers [63]. These general risks should be taken into account when considering surgical treatment, especially in the treatment of elderly patients as they are prone to increased risks of postoperative complications [64].

Unfortunately, there is a lack of clinical trials assessing the application of OSA therapies in edentulous patients. In order to improve clinical management, future studies in the form of randomized controlled trials (RCT) that assess the potential of implant-retained MAD therapy and other inventions are crucial. Adequate treatment is of key importance in an effort to reduce potentially serious complications and improve both the overall health and quality of life of these usually elderly patients [3, 65, 66].

## Conclusion

Currently, there is no consensus on the effects of nocturnal denture wearing on OSA. The use of modified MADs in edentate patients has been reported successful in selected cases. By avoiding poor retention, implant-retained MADs seem to be a viable treatment alternative to CPAP in the treatment of edentulous OSA patients. Few studies demonstrated the outcomes from surgical treatment in edentulous OSA patients. The overview of the literature presented in this paper demonstrates the paucity of effective evidence-based therapeutic strategies for edentulous OSA patients and the need for further clinical studies to improve clinical management.
